# Endoplasmic reticulum chaperone GRP78 is involved in autophagy activation induced by ischemic preconditioning in neural cells

**DOI:** 10.1186/s13041-015-0112-3

**Published:** 2015-03-26

**Authors:** Xiang-Yang Zhang, Tong-Tong Zhang, Dan-Dan Song, Jun- Hao Zhou, Rong Han, Zheng-Hong Qin, Rui Sheng

**Affiliations:** Department of Pharmacology and Laboratory of Aging and Nervous Diseases, Soochow University School of Pharmaceutical Science, 199 Ren Ai Road, Suzhou, 215123 China

**Keywords:** GRP78, Autophagy, PC12 cells, Cortical neurons, Ischemic preconditioning, BAPTA, RNA interference, Lentiviral vector, AMPK, mTOR

## Abstract

**Background:**

Our previous finding showed that brain ischemic preconditioning mediates neuroprotection through endoplasmic reticulum (ER) stress-induced autophagy. This study was aimed at exploring the role of ER chaperone GRP78 in IPC induced autophagy activation in neural cells.

**Results:**

Ischemic preconditioning (IPC) and oxygen glucose deprivation (OGD) models were established in rat pheochromocytoma (PC12) cells and primary cultured murine cortical neurons. IPC exerted neuroprotection against subsequent OGD injury in both PC12 cells and primary cortical neurons. IPC increased GRP78 expression and activated autophagy, as evidenced by upregulated LC3 and Beclin1, increased autophagic flux and formation of autophagosomes. BAPTA(dibromo-1,2-bis(aminophenoxy)ethane N,N,N9,N9 - tetra acetic acid, 0.125-2 μM) and small interfering RNA targeted GRP78 abrogated IPC induced neuroprotection and decreased the expression of GRP78, LC3II/LC3I and Beclin1. In contrast, lentiviral vector mediated GRP78 overexpression (LV-GRP78) strengthened resistance of PC12 cells to OGD injury and increased LC3 and Beclin1 expression. Moreover, knockdown of GRP78 in stable GRP78 overexpressing PC12 cells abolished the upregulation of LC3II/LC3I. GRP78 might activate autophagy through AMPK - mTOR pathway.

**Conclusion:**

These results suggest that IPC- induced GRP78 upregulation is involved in autophagy activation, and hence exerts protection against ischemic injury in neural cells.

**Electronic supplementary material:**

The online version of this article (doi:10.1186/s13041-015-0112-3) contains supplementary material, which is available to authorized users.

## Background

Ischemic preconditioning (IPC) is a sublethal ischemic episode, which initiates endogenous cytoprotective mechanisms to protect cells from subsequent severe ischemia [1]. Uncovering the mechanisms of brain ischemic preconditioning might lead to the development of effective treatments for ischemic cerebrovascular disease that could be exploited therapeutically [2,3]. The mechanisms of IPC have been systematically studied and several molecular pathways participating in preconditioning have been discovered, but definitive mechanisms underlying IPC remain incompletely understood to date [4-7].

Autophagy is an evolutionarily conserved and lysosome dependent protein degradation pathway [8-10]. Previous results, including our own, found that autophagy activation during IPC offers remarkable tolerance against subsequent fatal ischemic insult [11-13]. We also demonstrated that endoplasmic reticulum (ER) stress-induced autophagy is involved in the neuroprotection of ischemic preconditioning in vivo [14].

The ER facilitates the proper folding of newly synthesized proteins destined for secretion and provides the cell with a Ca^2+^ reservoir [15,16]. Cellular stress conditions, such as glucose deprivation, depletion of ER Ca^2+^ stores, exposure to free radicals, and accumulation of unfolded/misfolded proteins disrupt the proper function of the ER and initiate the unfolded protein response (UPR) [17-20]. One of the most important protective mechanisms induced by UPR is the upregulation of glucose regulated protein 78 (GRP78) [18,19]. GRP78, also known as the immunoglobulin heavy chain binding protein (BiP), is a member of the heat shock protein 70 (Hsp70) family, whose function is to maintain cytosolic homeostasis [21]. As a molecular chaperone, GRP78 regulates protein folding and facilitates protein translocation in the ER and it is also involved in ER stress [22]. Hypoxia, glucose deprivation, inflammation and Ca^2+^ stress can promote GRP78 mRNA transcription and translation to maintain the stability of the ER and to relieve severe ER stress. Recent reports demonstrated that GRP78 is related to autophagy activation in some tumor cell lines [23-25]. GRP78 knockdown leads to ER expansion and blocks the formation of autophagosomes induced by ER stress [23]. The GRP78 N-terminal ATPase domain folds into four discrete lobes, while GRP78 lobe IIB, consisting of amino-acid residues 236–314, represents the minimal binding region with insulin-like growth factor binding protein-3 (IGFBP-3) and contributes to autophagy activation [25]. In addition, GRP78 is involved in therapeutic resistance of antiestrogen-resistance breast cancer [24]. GRP78 overexpression increases the formation of autophagosomes, while GRP78 siRNA silencing decreases cell density accompanied by inhibition of autophagy, indicating that GRP78 is necessary for autophagy initiation in cancer. We thus hypothesized that GRP78 is involved in autophagy activation during brain ischemic preconditioning and, subsequently, contributes to ischemic tolerance.

In this study, we established the models of ischemic preconditioning (IPC) and oxygen glucose deprivation (OGD) in PC12 cells and primary cultured murine cortical neurons to explore the internal relation between ER molecular chaperon GRP78 and autophagy activation in preconditioning.

## Results

### Ischemic preconditioning (IPC) upregulated GRP78, induced autophagy and protected PC12 cells from OGD injury

**Figure 1:**
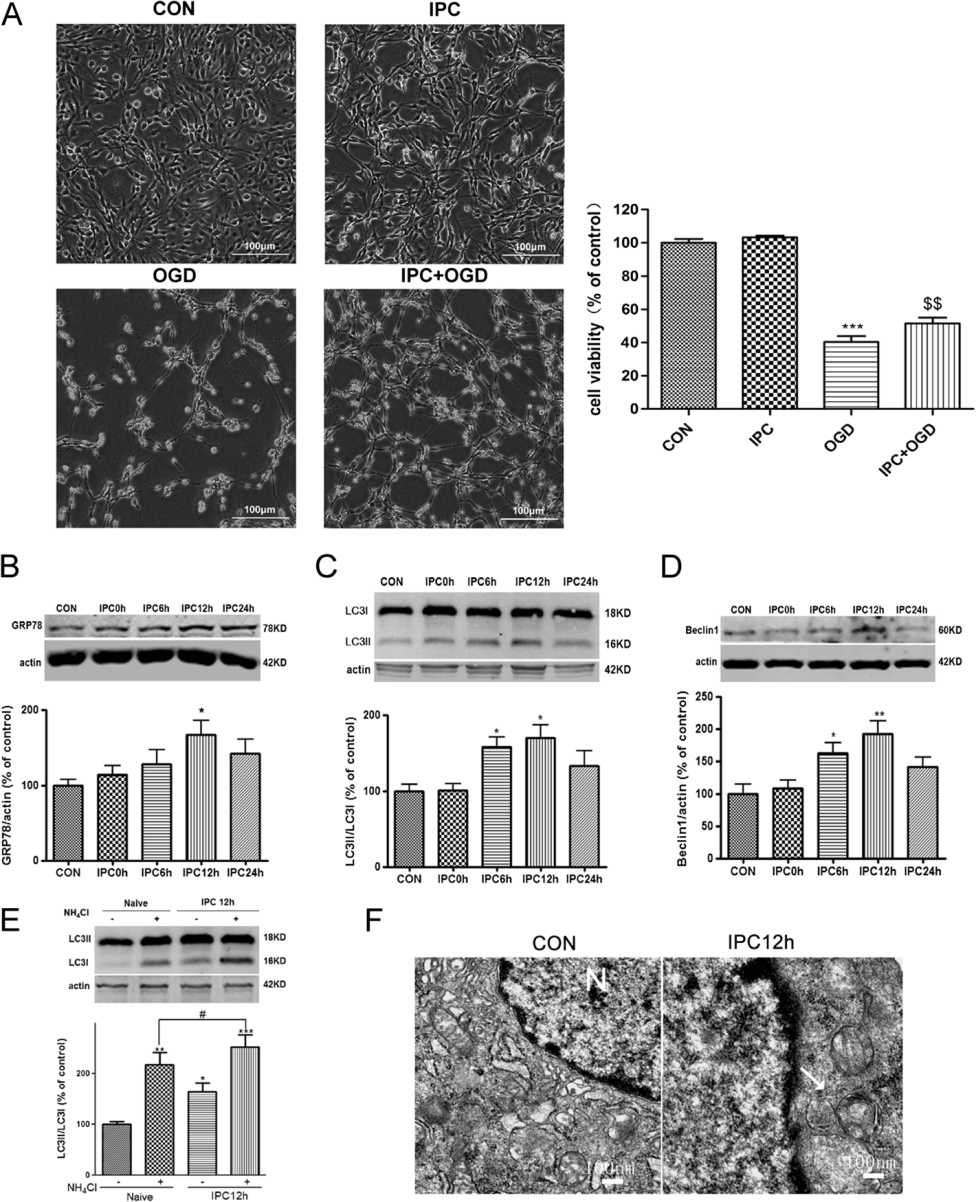
**Ischemic preconditioning (IPC) upregulated GRP78 and induced autophagy in PC12 cells. (A)** PC12 cells were exposed to oxygen glucose deprivation (OGD) for 30 min to induce IPC. Twelve hours after IPC, the cells were subjected to OGD for 10 h. The cell viability was examined with cell counting kit-8 (CCK8) and an optical microscope. Scale bar = 100 μm. **(B)-(D)** The cells were harvested 0, 6, 12 and 24 h after IPC. **(B)** GRP78 was upregulated after IPC. **(C)** LC3II/LC3I was upregulated after IPC. **(D)** Beclin1 was upregulated after IPC. **(E)** Autophagic flux was examined by comparing accumulation of LC3-II with and without NH_4_Cl. NH_4_Cl 20 mM treatment was given during the IPC episode. Cells were harvested at 12 h after IPC. Bar represents mean ± SD, n = 3. * *P* < 0.05, ** *P* < 0.01, ***P < 0.001 compared with the control group; $$ *P* < 0.01 compared with the OGD group. **(F)** Representative electron microscopic images show increased number of double-membrane vacuolar structures in PC12 cells after IPC. PC12 cells were harvested at 12 h after IPC. The cells were fixed in 0.25% glutaradehyde and examined with a transmission electron microscope. N: nucleus. Arrows indicate typical autophagosomes. Insets in the pictures were enlarged autophagosomes taken from the areas indicated by arrows. Scale bar = 100 nm.

We first determined whether IPC (a brief OGD of 30 min) induced ischemic tolerance against subsequent lethal OGD (10 h) in PC12 cells. Lethal OGD for 10 h induced significant cell injury as evidenced by morphological observation and CCK8 assay (*P* < 0.001 *v.s.* the control group; Figure 1A), while IPC greatly attenuated OGD induced cellular damage (*P* < 0.01 *v.s.* the OGD group). Then we examined GRP78 expression and autophagy activity in PC12 cells at different time points after IPC. GRP78 was upregulated after IPC and the peak GRP78 level was seen 12 h after IPC (Figure 1B, *P* < 0.05 *v.s.* the control group). Activation of autophagy was examined by immunoblotting of LC3 and Beclin1 [26,27]. Our results showed that LC3II/LC3I ratio and Beclin1 were increased after IPC (Figure 1C, D, *P* < 0.05 or *P* < 0.01 *v.s.* the control group), with maximal effects observed at 12 h after IPC. To further confirm that IPC can induce autophagic flux, we then analyzed LC3-II levels after IPC with ammonium chloride (NH_4_Cl) treatment, which could neutralize the acidic pH to block lysosome degradation [28,29]. Treatment with NH_4_Cl alone causes accumulation of LC3-II(*P* < 0.01 *v.s.* the control group, Figure 1E), but IPC+ NH_4_Cl further enhanced the accumulation of LC3-II (*P* < 0.05 *v.s.* the IPC group), indicating that IPC stimulates autophagic flux. LC3 and GRP78 upregulation at 12 h after IPC was confirmed with immunofluorescence (Additional file 1: Figure S1). LC3 was not co-localized with GRP78 in control but the two were highly co-localized at 12 h after IPC, suggesting that GRP78 might localize into the autophagosomes. The formation of autophagosomes was also observed under an electron microscope at 12 h after IPC (Figure 1F). Control PC12 cells appeared normal with relatively healthy-looking organelles and nuclei. Twelve hours after IPC, the organelles and nuclei in PC12 cells also seemed normal without appreciable injury, but more double-membrane or multi-membrane vacuolar structures were found, suggesting possible autophagy induction after IPC. All these results indicate that ischemic preconditioning increases GRP78 expression and upregulates autophagy in PC12 cells.

### Suppression of GRP78 inhibited autophagy activation and neuroprotection induced by IPC

**Figure 2:**
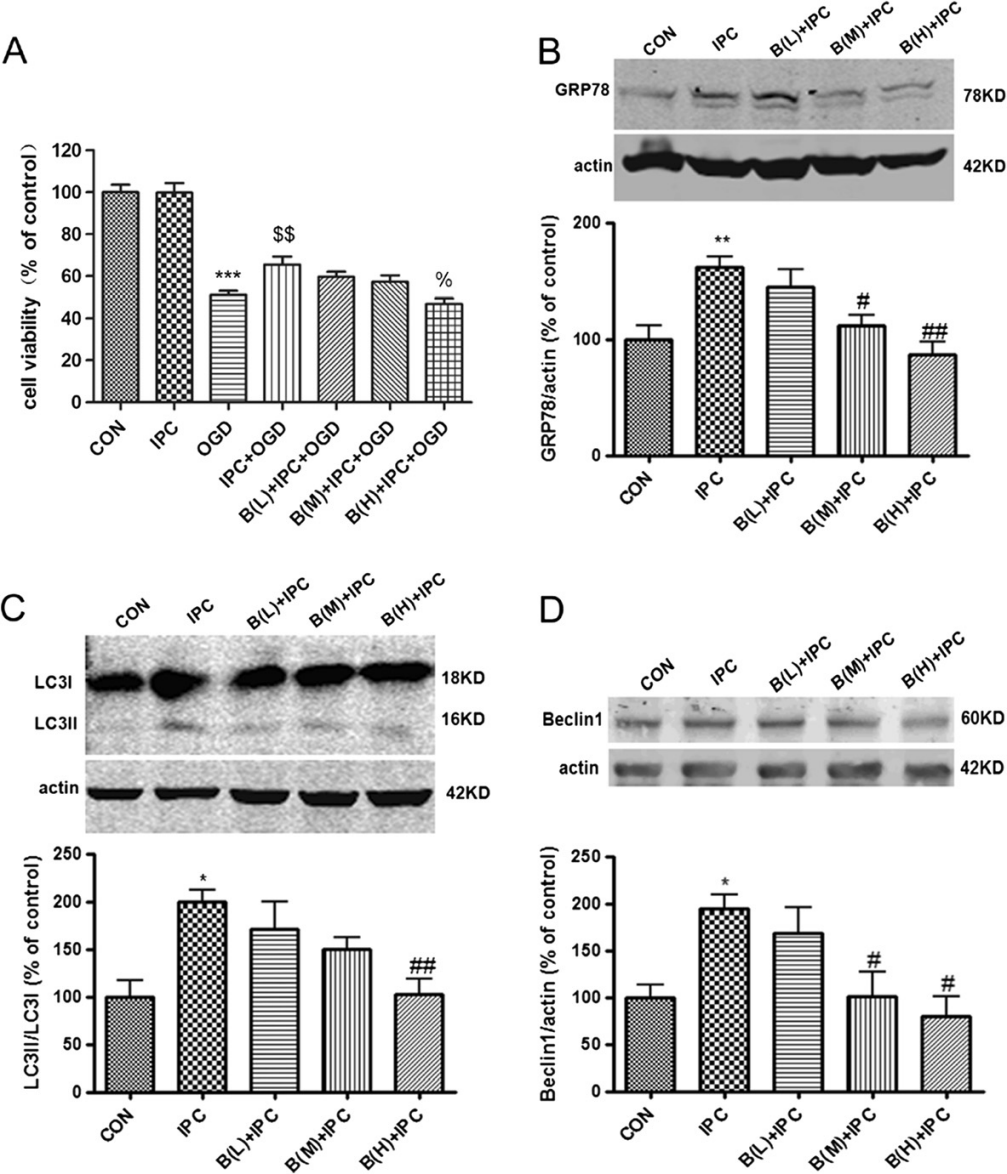
**BAPTA inhibited autophagy activation and neuroprotection induced by IPC in PC12 cells. (A)** BAPTA abolished IPC induced neuroprotection in PC12 cells. Cells were incubated with BAPTA 60 min before the onset of IPC. Twelve hours after IPC, the cells were subjected to OGD for 10 h and cell viability was examined with CCK-8 kit. **(B)-(D)** The cells were incubated with BAPTA 60 min before the onset of IPC. Then the cells were harvested 12 h after IPC and subjected to Western blot analysis. **(B)** BAPTA reduced the expression of GRP78 protein. **(C)** BAPTA reduced LC3II/LC3I ratio. **(D)** BAPTA reduced the expression of Beclin1 protein. Bar represents mean ± SD, n = 3. *P < 0.05, **P < 0.01, ***P < 0.001 compared with the control group; $$ *P* < 0.01 compared with the OGD group; % *P* < 0.05 compared with the IPC + OGD group; # *P* < 0.05, ##*P* < 0.01 compared with the IPC group. B (L) = BAPTA 0.125 μM; B (M) = BAPTA 0.5 μM; B (H) = BAPTA 2 μM.

To investigate the role of GRP78 in IPC’s neuroprotection against OGD injury, we used BAPTA, a membrane-permeable Ca^2+^ chelator, which non-specifically suppresses GRP78 expression in a variety of cell lines [30,31]. Our results showed that BAPTA (0.125-2 μM) had no effects on the cell viability of PC 12 cells when treating the cells for 48 h (data not shown). Lethal OGD for 10 h induced significant cell injury in PC12 cells (*P* < 0.001 *v.s.* the control group; Figure 2A). IPC greatly attenuated lethal OGD-induced cell injury (*P* < 0.01 *v.s.* the OGD group), whereas BAPTA 2 μM pretreatment partly recovered the OGD-induced cellular damage (*P* < 0.05 *v.s.* the IPC + OGD group). To examine whether BAPTA inhibits GRP78 expression and blocks the autophagy activation after IPC, we examined the protein levels of GRP78, LC3 and Beclin1 in PC12 cells after BAPTA treatment. Western blot analysis revealed that GRP78 was upregulated in IPC group (Figure 2B; *P* < 0.01 *v.s.* the control group), while BAPTA attenuated IPC elicited GRP78 upregulation (*P* < 0.05 or *P* < 0.01 *v.s.* the IPC group), suggesting that BAPTA suppressed IPC-induced GRP78 upregulation. The LC3-II and Beclin1 levels were also significantly upregulated after IPC, while the induction of LC3-II and Beclin1 by preconditioning was markedly blunted by BAPTA (*P* < 0.05 or *P* < 0.01 *v.s.* IPC group, Figure 2C, D).

**Figure 3:**
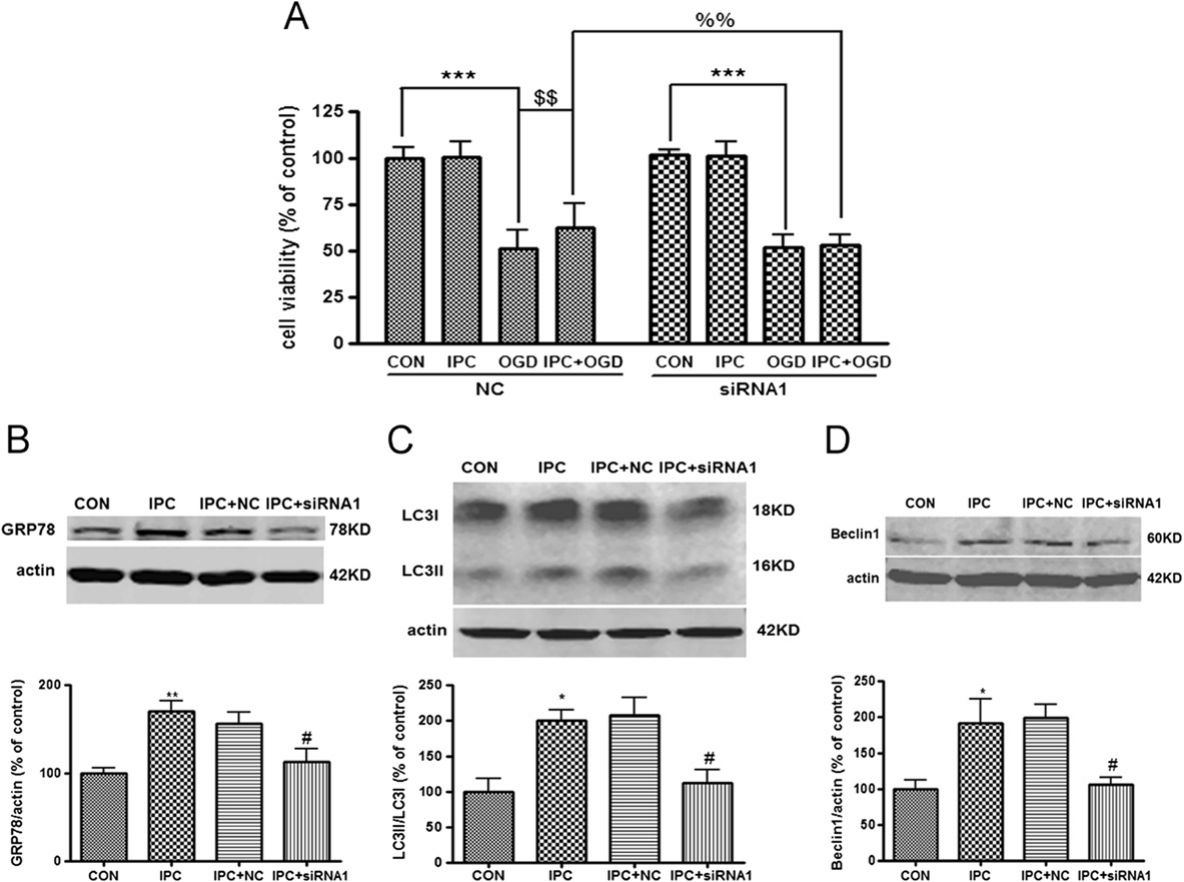
**Suppression of GRP78 with siRNA inhibited autophagy activation and neuroprotection induced by IPC in PC12 cells. (A)** GRP78 siRNA1 cancelled the IPC-induced neuroprotection. Cells were transfected with GRP78 siRNA1 (40 nM) or negative control sequence (NC) twenty-four hours before the onset of IPC. Twelve hours after IPC, the cells were subjected to OGD for 10 h and cell viability was examined with CCK-8 kit. **(B)-(D)** The cells were harvested 12 h after IPC and subjected to Western blot analysis. **(B)** GRP78 siRNA1 reversed IPC-induced upregulation of GRP78. **(C)** GRP78 siRNA1 reversed IPC-induced upregulation of LC3II/LC3I. **(D)** GRP78 siRNA1 reversed IPC-induced upregulation of Beclin1. Bar represents mean ± SD, n = 3. *P < 0.05, **P < 0.01, ***P < 0.001 compared with the control group; $$*P* < 0.01 compared with the OGD group; %% *P* < 0.01 compared with the NC + IPC + OGD group; # *P* < 0.05 compared with the NC + IPC group.

Since BAPTA is not a GRP78 specific inhibitor, we then transfected PC12 cells with siRNAs targeting GRP78 or control siRNA. Transfection of PC12 cells with two GRP78 siRNAs resulted in a marked decrease in GRP78 expression compared with negative control siRNA (NC) in the absence or presence of IPC (*P* < 0.05 *v.s.* NC, Additional file 2: Figure S2A, B, Figure 3B). In addition, LC3II/LC3I conversion and Beclin1 protein level were also downregulated in GRP78 siRNAs-transfected cells (*P* < 0.05 *v.s.* NC + IPC, Figure 3C, D, Additional file 2: Figure S2C). Importantly, depletion of GRP78 by GRP78 siRNA1 apparently abolished ischemic tolerance induced by IPC (*P* < 0.01 *v.s.* NC + IPC + OGD, Figure 3A).

**Figure 4:**
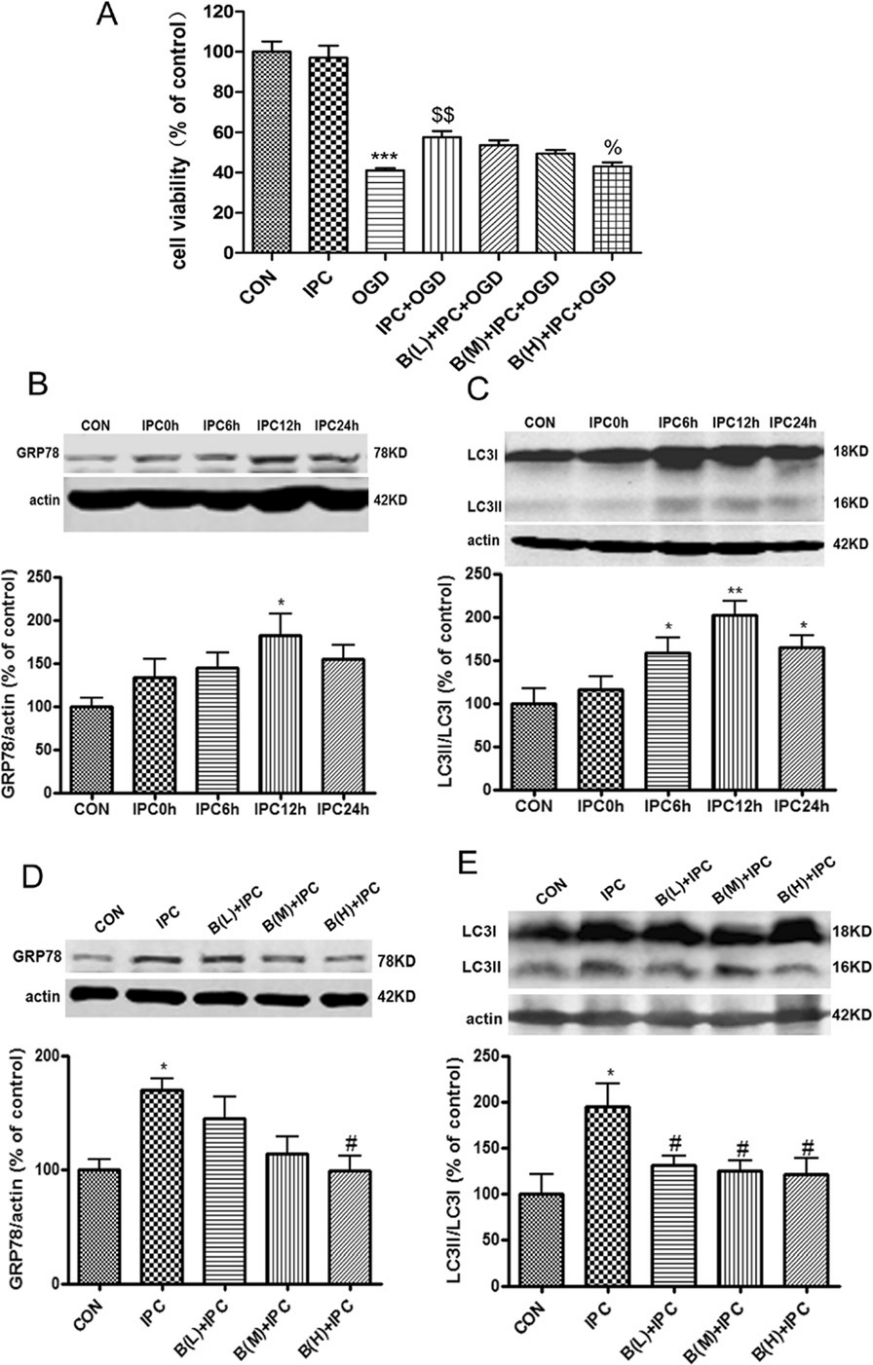
**BAPTA inhibited autophagy activation and neuroprotection induced by IPC in primary cultured cortical neurons. (A)** Cortical neurons were exposed to OGD for 30 min to induce IPC. Twenty-four hours after IPC, the neurons were subjected OGD for 4 h. The cell viability was examined with CCK8. **(B)**, **(C)** The cells were harvested 0, 6, 12 and 24 h after IPC. **(B)** GRP78 was upregulated after IPC. **(C)** LC3II/LC3I was upregulated after IPC. **(D)**, **(E)** The neurons were incubated with BAPTA 60 min before the onset of IPC and harvested 12 h after IPC. **(D)** BAPTA reduced IPC-induced upregulation of GRP78. **(E)** BAPTA reduced IPC-induced upregulation of LC3II/LC3I. Bar represents mean ± SD, n = 3. *P < 0.05, **P < 0.01, ***P < 0.001 compared with the control group; $$ *P* < 0.01 compared with the OGD group; % *P* < 0.05 compared with the IPC + OGD group; # *P* < 0.05 compared with the IPC group. B (L) = BAPTA 0.125 μM; B (M) = BAPTA 0.5 μM; B (H) = BAPTA 2 μM.

We also confirmed parts of the above results in primary cultured murine cortical neurons. Lethal OGD for 4 h induced significant cell injury in cortical neurons (*P* < 0.001 *v.s.* the control group, Figure 4A), while IPC effectively attenuated OGD induced neuronal injury (*P* < 0.01 *v.s.* the OGD group). However, BAPTA 2 μM abolished the IPC induced ischemic tolerance (*P* < 0.05 *v.s.* the IPC + OGD group). Similar to PC12 cells, GRP78 levels and LC3II/LC3I ratio were increased in cortical neurons after IPC (*P* < 0.05 or *P* < 0.01 *v.s.* the control group, Figure 4B, C), with maximal effects appearing at 12 h after IPC, while BAPTA induced a decline of GRP78 and LC3-II/LC3I (*P* < 0.05 *v.s.* the IPC group, Figure 4D, E).

All these results suggest that suppression of GRP78 inhibits autophagy activation and neuroprotection induced by ischemic preconditioning in both PC12 cells and cortical neurons.

### GRP78 overexpression induced autophagy activation and strengthened cells’ resistance against OGD

**Figure 5:**
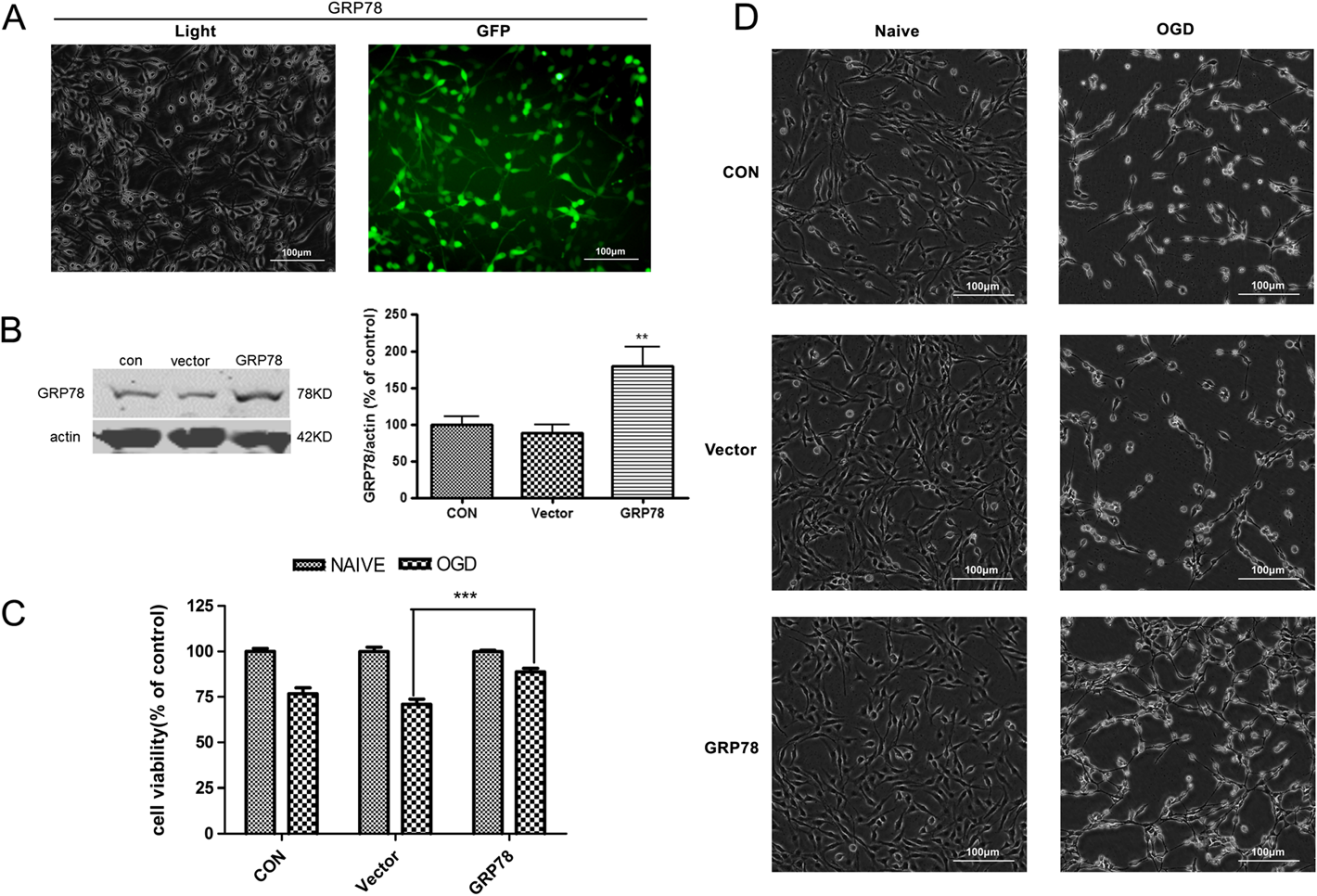
**GRP78 overexpression strengthened resistance of PC12 cells to OGD. (A)** The LV-GRP78-PC12 cells were observed under a light (left) and fluorescent (right) microscope, scale bar = 100 μm. **(B)** The GRP78 expression increased in the LV5- GRP78-PC12 cells. The control, LV-vector, LV-GRP78-PC12 were harvested and subjected to Western blotting analysis. **(C)**, **(D)** Control, LV-vector, LV-GRP78-PC12 were exposed to OGD for 10 h. The cell viability was examined with CCK8 **(C)** and an optical microscope **(D)**. The bar represents the ratio of the OGD groups of control, LV-vector, LV-GRP78-PC12 cells versus their respective controls. Bar represents mean ± SD, n = 3. **P < 0.01, ***P < 0.001 compared with LV- vector.

**Figure 6:**
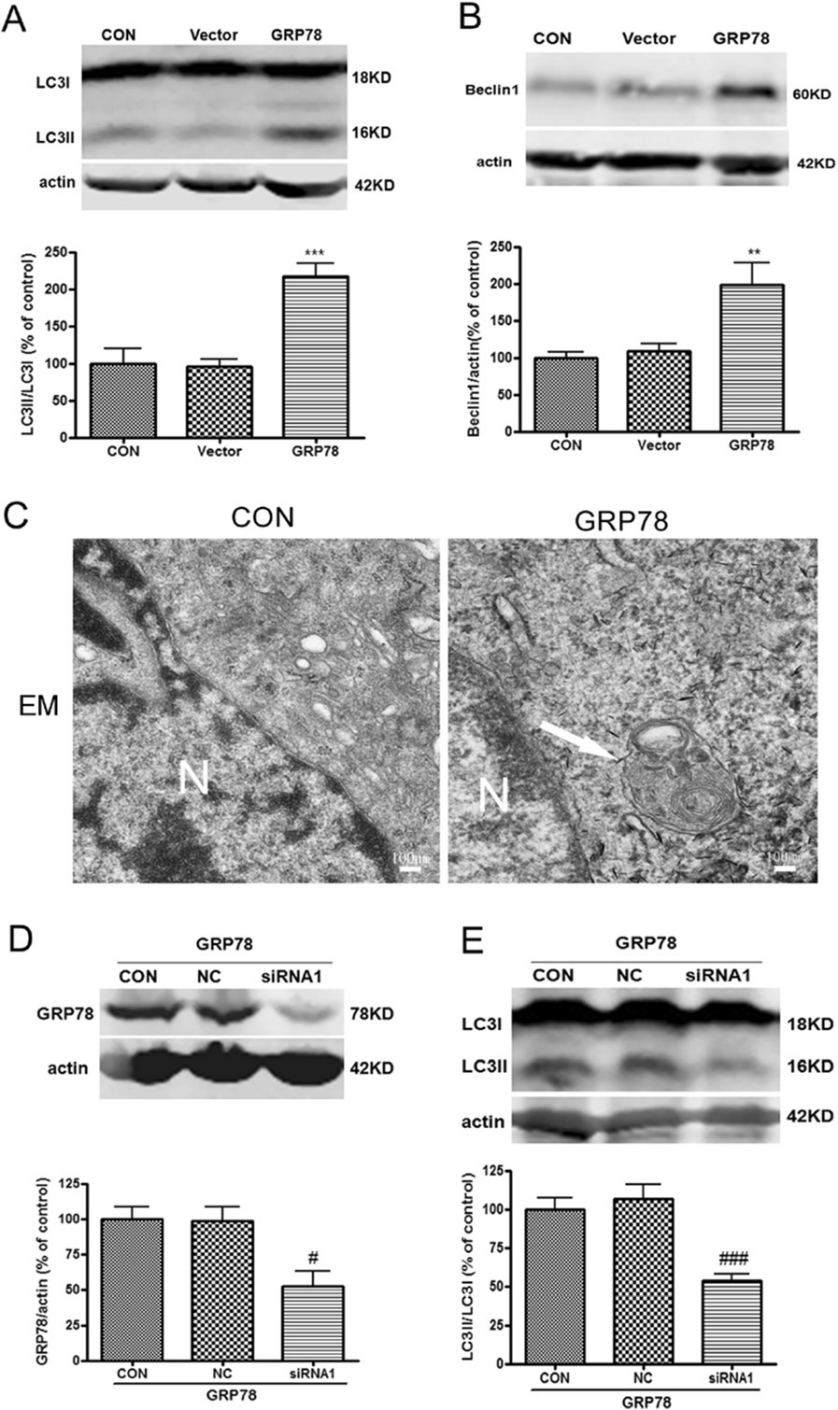
**GRP78 overexpression induced autophagy activation in PC12 cells. (A)**, **(B)**: The control, LV-vector, LV-GRP78-PC12 cells were harvested and subjected to Western blotting analysis. **(A)** LC3II/LC3I was upregulated in LV-GRP78-PC12 cells. **(B)** Beclin1 was upregulated in LV-GRP78-PC12 cells. **(C)** Electron microscopic images show increased number of double-membrane vacuolar structures in the LV-GRP78-PC12 cells. The cells were fixed in 0.25% glutaradehyde and examined with a transmission electron microscope. N: nucleus. Arrows indicated typical autophagosomes. Scale bar = 100 nm. **(D)**, **(E)** LV-GRP78-PC12 cells were transfected with GRP78 siRNA1 (40 nM) or negative control sequence (NC). Forty-eight hours later, the cells were harvested and subjected to Western blotting analysis. **(D)** GRP78 siRNA1 reduced GRP78 expression. **(E)** GRP78 siRNA1 reduced LC3II/LC3I. Bar represents mean ± SD, n = 3. **P < 0.01, ***P < 0.001 compared with LV- vector; # *P* < 0.05, ### *P* < 0.001 compared with NC.

To further validate the proposal that GRP78 contributes to autophagy activation during preconditioning, we generated GRP78 stable overexpressing cell lines (LV-GRP78-PC12 cells) by transfecting GRP78 lentivirus into parental PC12 cells. We observed the GFP green fluorescence and found that >90% of cells expressed GFP fluorescence in LV-GRP78-PC12 cells (Figure 5A). GRP78 level in LV-GRP78-PC12 cells was significantly higher than LV-vector-PC12 cells (*P* < 0.01 *v.s.* the LV-vector, Figure 5B). Then we examined whether GRP78 overexpression strengthened cells’ resistance against OGD. The results showed that OGD-induced cell death in GRP78 stable expressing cells was significantly lessened as evidenced by morphological observation and CCK8 assay (*P* < 0.001 compared with LV-vector transfected PC12 cells, Figure 5C, D), suggesting that GRP78 overexpression protects cells against OGD injury. LC3II/LC3I and Beclin1 expression were also upregulated in LV-GRP78-PC12 cells (*P* < 0.01 or *P* < 0.001 *v.s.* the LV-vector, Figure 6A, B). Autophagy activation was also confirmed by the increased formation of autophagosomes observed under an electron microscope (Figure 6C). We further depleted GRP78 in stable GRP78 expressing PC 12 cells by siRNA transfection. In GRP78 overexpressing cells, knockdown of GRP78 significantly decreased GRP78 and LC3II protein levels (*P* < 0.05 or *P* < 0.001 *v.s.* the NC group, Figure 6D, E). All these results demonstrated that GRP78 overexpression induced autophagy activation in PC12 cells and strengthened cells’ resistance against OGD.

### GRP78 may induce autophagy activation through AMPK-mTOR pathway

**Figure 7:**
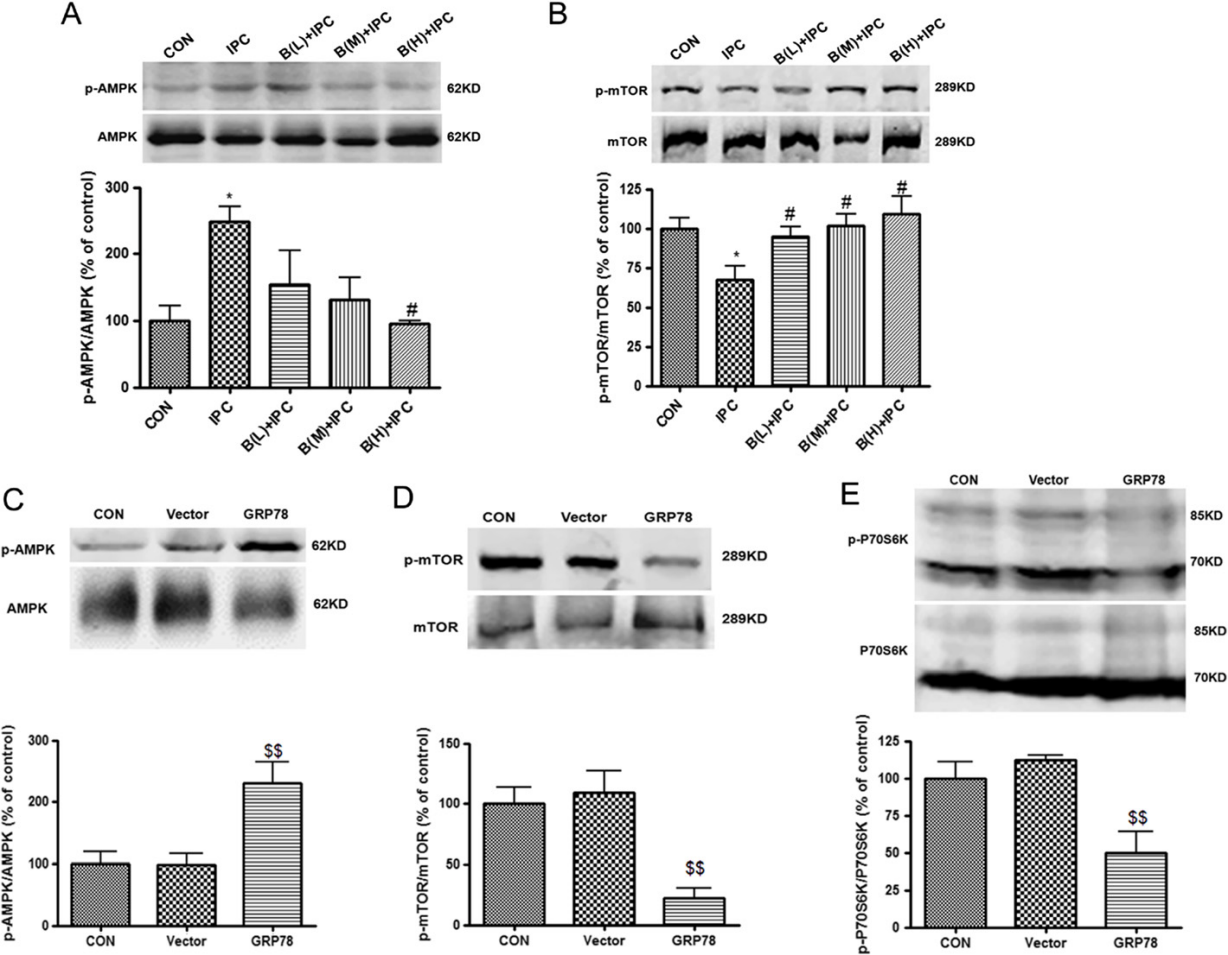
**GRP78 may activate autophagy through AMPK-mTOR pathway. (A)**, **(B)** The PC12 cells were incubated with BAPTA 60 min before the onset of IPC. Then the cells were harvested 12 h after IPC and subjected to Western blotting analysis. **(A)** BAPTA reduced p-AMPK/AMPK ratio. **(B)** BAPTA increased p-mTOR/mTOR ratio. B (L) = BAPTA 0.125 μM; B (M) = BAPTA 0.5 μM; B (H) = BAPTA 2 μM. **(C)**-**(E)** The control, LV-vector, LV-GRP78-PC12 cells were harvested and subjected to Western blotting analysis. **(C)** p-AMPK/AMPK ratio was upregulated in LV-GRP78-PC12 cells. **(D)** p-mTOR/mTOR ratio was downregulated in LV-GRP78-PC12 cells. **(E)** p-P70S6K/P70S6K ratio was downregulated in LV-GRP78-PC12 cells. n = 3. *P < 0.05 compared with the control group; # *P* < 0.05 compared with the IPC group; $$ *P* < 0.01 compared with LV- vector.

**Figure 8:**
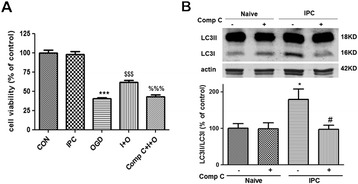
**AMPK inhibition by compound C inhibited autophagy activation and neuroprotection induced by IPC in PC12 cells. (A)** Compound C (Comp C) abolished IPC induced neuroprotection in PC12 cells. Cells were incubated with compound C 5 μM 60 min before the onset of IPC. Twelve hours after IPC, the cells were subjected to OGD for 10 h and cell viability was examined with CCK-8 kit. **(B)** Compound C reduced LC3II/LC3I ratio. The cells were incubated with compound C 5 μM 60 min before the onset of IPC. Then the cells were harvested 12 h after IPC and subjected to Western blot analysis. Bar represents mean ± SD, n = 3. *P < 0.05, ***P < 0.001 compared with the control group; $$$ *P* < 0.001 compared with the OGD group; %%% *P* < 0.001 compared with the IPC + OGD group; # *P* < 0.05 compared with the IPC group.

To further investigate the potential mechanisms by which GRP78 mediates autophagy, we examined phosphorylation of AMPK and mTOR in the case of GRP78 suppression and overexpression. The results showed that in IPC group, the phosphorylation of AMPK was enhanced, while the phosphorylation of mTOR was reduced compared with the control group (*P* < 0.05, Figure 7A, B), but BAPTA seemed to inhibit the changes in ratios of p-AMPK/AMPK and p-mTOR/mTOR induced by IPC (*P* < 0.05 *v.s.* IPC group), suggesting that AMPK - mTOR pathway might be involved in GRP78 induced autophagy during IPC. In addition, GRP78 overexpression induced phosphorylation of AMPK, while inhibiting the induction of p-mTOR and p-P70S6K (*P* < 0.01 *v.s.* the LV-vector, Figure 7C, D, E). To determine if AMPK links GRP78 or IPC to autophagy activation, we employed compound C, which functions as an ATP-competitive inhibitor of AMPK [32,33]. Treatment with compound C abolished the IPC induced ischemic tolerance (Figure 8A, *P* < 0.001 *v.s.* IPC + OGD group), and decreased the LC3II/LC3I ratio (Figure 8B, *P* < 0.05 *v.s.* IPC group), suggesting that AMPK is required for autophagy activation in response to IPC. These results, combined with the fact that suppression or overexpression of GRP78 induces corresponding changes in AMPK - mTOR pathway, support the hypothesis that GRP78 may activate autophagy through AMPK-mTOR pathway.

## Discussion

Our previous studies suggested that ER stress might contribute to autophagy activation and neuroprotection induced by ischemic preconditioning [12,14], but the exact mechanism by which ER stress activates autophagy has not been fully elucidated. During ER stress, ER molecular chaperone GRP78 dissociates from the ER transmembrane signal transduction proteins to promote the proper folding of unfolded proteins and regulate intracellular calcium balance [22,23,34]. Induction of GRP78 was also shown to prevent neuronal death during ischemia both in vitro and in vivo [35-37]. We thus assume that the ER chaperon GRP78 might be the key regulator contributing to autophagy activation during preconditioning.

The highly differentiated rat pheochromocytoma (PC12) cells share many typical properties with neuronal cells, including outgrowth of neurites, synthesis of neurotransmitters, selective expression of certain proteins and interactions of compounds with membrane bound receptors [38-40]. Thus, in the present study, we first used the PC12 cell lines to established IPC (a brief OGD of 30 min) and OGD (10 h) models. The GRP78 expression and autophagy activity at different time points after IPC were examined by application of multiple detection methods involving Western blot analysis, immunofluorescence and electron microscopy. We found that lethal OGD induced significant cell injury, while IPC greatly attenuated OGD- induced cellular damage. IPC upregulated GRP78, LC3II/LC3I ratio, and Beclin1 expression while also increasing autophagic flux and formation of autophagosomes. GRP78, LC3II/LC3I ratio and Beclin1 all peaked at 12 h after IPC. These results suggest that ischemic preconditioning increases levels of ER molecular chaperon GRP78 and induces autophagy simultaneously.

To investigate the role of GRP78 in ischemic tolerance and autophagy activation, we suppressed GRP78 expression with BAPTA, a Ca^2+^ chelator as well as a nonspecific GRP78 inhibitor [30,31], and siRNAs targeting GRP78. Our results showed that both BAPTA and GRP78 siRNAs attenuated IPC elicited GRP78 upregulation. In addition, LC3 II/LC3 I conversion and Beclin1 protein were also downregulated with BAPTA and GRP78 siRNAs. Importantly, depletion of GRP78 apparently abolished ischemic tolerance induced by IPC. The above results found in PC12 cells were also partly confirmed in primary cultured murine cortical neurons. IPC increased GRP78 protein expression and activated autophagy simultaneously in neurons, in consistence with our previous finding [13]. Furthermore, suppression of GRP78 with BAPTA abolished the neuroprotection of IPC, and inhibited IPC-induced autophagy activation. All these results indicate that suppression of GRP78 inhibits autophagy activation and neuroprotection induced by ischemic preconditioning in both PC12 cells and cortical neurons.

To further validate the proposal that GRP78 contributes to autophagy activation during preconditioning, we generated GRP78 stable overexpressing cell lines (LV-GRP78-PC12 cells) by transfecting GRP78 lentivirus into parental PC12 cells. Our results showed that GRP78 overexpression significantly lessened OGD- induced cell death in PC12 cells. LC3II/LC3I and Beclin1 expression were upregulated in LV-GRP78-PC12 cells and increased formation of autophagosomes was also observed. When we further depleted GRP78 in stable GRP78 expressing PC 12 cells, knockdown of GRP78 significantly decreased autophagy activity. All these results demonstrated that GRP78 overexpression and autophagy activation might mimic the neuroprotection of IPC to strengthen cells’ resistance against OGD.

Actually autophagy plays divergent roles during preconditioning and ischemia. Some studies even revealed the distinct roles of autophagy during ischemia and reperfusion [41]. Our lab had found that inhibition of autophagy in the permanent ischemic brain with 3-MA attenuates the ischemic injury in rats [42]. By contrast, autophagy might contribute to neuroprotection during reperfusion and IPC episode. Inhibition of autophagy with 3-MA during the reperfusion phase after OGD enhanced cell death [41] and pretreatment with 3-MA before IPC abolished the IPC tolerance [12,13]. Thus we believe that induction of GRP78 or autophagy before ischemia might mimic preconditioning effects to exert protection against ischemic stroke.

To investigate the potential mechanisms by which GRP78 mediates autophagy, we examined the AMPK-mTOR pathway in further experiments. AMPK (AMP activated protein kinase) is an evolutionally conserved eukaryotic protein kinase perceiving the cellular energy status. In case of energetic stress or low energy (AMP/ATP ratio increases), AMPK is activated to regulate the energy balance through diverse cellular pathways [43]. During cardiac ischemia, AMPK enhances glucose transport and glycolysis [44]. AMPK also plays an important role in IPC-induced cardioprotection by promoting GLUT4 translocation and facilitating glucose uptake [45]. Knockdown of GRP78 in ovarian cancer cells cancelled the induction of autophagy and UPR induced by diindolylmethane and AMPK was found to be involved in diindolylmethane initiated autophagy [46]. Mounting evidence shows that AMPK activates autophagy through inhibition of the target of rapamycin (TOR) [47-49]. When nutrients are abundant, mTOR (mammalian target of rapamycin) is active for maintaining cell growth. However, when rapidly growing cells are treated with rapamycin, hypoxia or amino starvation, the protein synthesis is generally downregulated and autophagy is activated through the Atg1 dependent pathway [50-52]. p70S6K1 (p70 ribosomal protein S6 kinases 1), a kind of ribosomal protein kinase, controls cell growth and migration. p70S6K1 can be activated through direct phosphorylation of Thr389 by mTOR, and thus the phosphorylation levels of p70S6K1 is often used to monitor mTOR activation [53]. Our results showed that in IPC group, the phosphorylation of AMPK was enhanced, while phosphorylation of mTOR was reduced, but BAPTA seemed to decrease p-AMPK/AMPK ratio and increase p-mTOR/mTOR ratio. On the contrary, GRP78 overexpression reduced the proportion of p - mTOR/mTOR and p-p70S6K/p70S6K, but increased the ratio of p-AMPK/AMPK. Accordingly, the inhibition of AMPK by a pharmacological inhibitor (compound C) attenuated autophagy and ischemic tolerance induced by IPC, indicating that IPC exerts its function on autophagy through activating AMPK. All these results support the proposition that GRP78 induced autophagy during IPC might be associated with AMPK - mTOR pathway, in agreement with the recent studies in cancer cells [24].

## Conclusions

In summary, our study found that ischemic preconditioning upregulated GRP78 expression and promoted autophagy in PC12 cells and cortical neurons. Specific suppression of GRP78 with pharmacological and genetic approaches inhibited autophagic activation and abolished ischemic tolerance. In addition, GRP78 overexpression activated autophagy and protected the cells from ischemic injury. Data suggest GRP78 may activate autophagy through AMPK-mTOR pathway. Our results also raise a possibility that drugs acting on GRP78-AMPK-mTOR-autophagy pathway might mimic preconditioning effects and be useful for the clinical treatment of ischemic stroke.

## Methods

### Cell culture

Highly differentiated rat PC12 cells were obtained from the Shanghai Institute of Cell Biology (Shanghai, China). Cells were cultured in Dulbecco’s modified Eagle’s medium (DMEM, Gibco) supplemented with 10% fetal bovine serum (FBS, Gibco), 100 mg/L streptomycin, 100U penicillin at 37°C in 5% CO_2_/95% air.

Primary neuronal culture was prepared from the cortex of E15-17 embryonic mice as described previously [13]. Care and handling of animals were approved by the Institutional Animal Care and Use Committee of Soochow University. The national guidelines for laboratory animal care and use were followed. Briefly, the timed pregnant mouse (E15-17, Center for Experimental Animals of Soochow University, certificate No 20020008, Grade II) was sacrificed under 2% isoflurane. The embryos were collected and their brains were harvested in sterile PBS. The cortices were dissected out into pre-cooled PBS, minced and digested with 2.5% trypsin solution at 37°C for 15 min. The sediments were then incubated with DMEM/F12 containing 10% fetal bovine serum and 0.5 mg/ml DNase for 3 min. The cell suspensions were disassociated by pipetting up and down and centrifuged at 500 × *g* for 5 min. The pellets were resuspended in Neurobasal medium (NBM, Invitrogen) containing 10% B27 (Gibco), 25 μM glutamate, 1% penicillin G and streptomycin. Then the cells were plated onto poly (D-lysine)-coated plates. Twenty-four hours later, the whole medium was replaced by NBM containing 10% B27, 0.5 mM glutamine, 1% penicillin G and streptomycin. Thereafter, half of the medium was replaced every 2 days. The cortical neurons matured at day 7 ~ 9d and then were used for further experiments.

### Oxygen glucose deprivation (OGD) and ischemic preconditioning (IPC) models

To induce OGD, the cultures were washed three times with Hepes balanced salt solution (HBSS: 140 mM NaCl, 3.5 mM KCl, 12 mM MgSO_4_, 5 mM NaHCO_3_, 1.7 mM CaCl_2_, 0.4 mM KH_2_PO_4_, 10 mM Hepes) and then placed in a modular incubator chamber (Billups-Rothenberg, MC-101) aerated with an anaerobic gas mixture (95% N_2_/5%CO_2_) [13]. To terminate OGD, cells were removed from the anaerobic chamber, and then were changed with normal medium. For IPC, the cells were deprived of oxygen and glucose for 30 min, an insult that did not induce neuronal death, as measured by cell viability assay in preliminary experiments. Twelve to twenty-four hours after IPC, the cultures were again deprived of oxygen and glucose for 10 h in PC12 cells and 4 h in cultured neurons to cause lethal OGD. The morphological changes of cells were observed using a phase contrast microscope.

### Cell viability assay

Cell viability was evaluated with the Cell Counting Kit-8 assay kit (CCK-8, Dojindo Laboratories, Kumamoto, Japan) following the manufacturer’s instruction. Briefly, the cells were seeded in 96-well cell culture plates. After treatment, the medium was changed to 100 μL normal medium, and 10 μL CCK-8 was added per well and incubated with the cells at 37°C for 2 h. The optical densities of samples were measured at 450 nm using a microplate reader (ELX 800, Bio-Tek). Every experiment was repeated three times.

### Western blotting

The cells were washed twice with ice-cooled PBS and lysed in a buffer containing Tris–HCl (pH 7.4) 10 mM, NaCl 150 mM, 1% Triton X-100, 1% sodium deoxycholate, 0.1% SDS, edetic acid 5 mM, and 1 protease inhibitor cocktail tablet (Roche)/10 ml. Protein concentrations were determined using a BCA kit (Pierce). A 30–50 μg aliquot of proteins from each sample was separated by SDS-PAGE and subsequently transferred to a nitrocellulose membrane. Afterwards, the membranes were incubated with the specific antibodies against LC3 (1:1000; Abcam, ab62721), GRP78 (1:500; Santa Cruz, sc-1050, Santa Cruz, CA, USA), Beclin1(1:500; Santa Cruz, sc-11427), phospho-mTOR (1:1000; Cell signaling, 2971), mTOR(1:1000; Cell signaling 2983), phospho-AMPKα(1:1000; Cell signaling, 2535), AMPK(1:1000; Cell signaling 2603), phospho-p70S6 kinase(1:1000; Cell signaling, 9206) or p70S6 kinase (1:1000; Abcam, ab32529) at 4°C for over night, then incubated with a secondary antibody (1:10000; LI-COR Biosciences, anti-Goat 926–32214; anti-Mouse 926–32212; anti-Rabbit 926–32213) at room temperature for 2 h. The membranes were then revealed by Odyssey Two-Color Infrared Imaging System (LI-COR, Lincoln, NE, USA) following the manufacturer’s instructions. The membranes were reprobed with β-actin (1:5,000; Sigma, A5441) after striping (TBST with 2% β-mecaptoethanol, 65°C, 1 h). The levels of interested proteins were analyzed with Sigma Scan Pro 5 and normalized to the loading control (β-actin).

### Immunofluorescence

The cells were grown onto poly (D-lysine)-coated cover glasses in a 24-well plate. After treatment, the cells were fixed for 20 min using cooled absolute ethyl alcohol, and then incubated with PBS containing 0.2% Triton X-100 for 30 min. After blocking with 5% non-fat milk in PBS for 1 h, the cells were then incubated with antibodies against GRP78 (1:500; Santa Cruz, sc-1050) or LC3 antibody (1: 400; MBL, PD 014) at 4°C for 24 h. The cells were sequentially incubated with Cy3-conjugated anti-goat IgG (1:400; Jackson ImmunoResearch, 705-166-147) and FITC-conjugated anti-rabbit IgG (1:400; Jackson ImmunoResearch, 111-095-003) for 2 h. Afterwards, the cells were incubated with 0.5 μg/ml 4,6-diamidino-2- phenylindole (DAPI, Sigma) for 10 min. Then the cells were mounted on slides and images of fluorescence were acquired using a laser confocal microscope (Carl Zeiss Microimage Inc., Thornwood, NY).

### Electron microscopy

Cells were harvested and fixed in 2.5% gluteraldehyde in 0.1 M phosphate buffer (pH 7.4) at room temperature. The cell pellets were post-fixed with 1% Osmium tetroxide, dehydrated through acetone and then immersed in resin. After hardening, the blocks were thinly sectioned at 50 nm thickness, stained with lead citrate and then viewed on a Philips CM-120 electron microscope.

### Drug treatment

PC12 cells or cortical neurons were pretreated with dibromo-1,2-bis(aminophenoxy) ethane N,N,N9,N9- tetraacetic acid (BAPTA, Invitrogen B1205) 0.125 μM, 0.5 μM, 2 μM or compound C (Dorsomorphin, Selleck S7306) 5 μM 60 min before IPC. The cells continued to be incubated with these agents during the reperfusion episode after IPC. To access autophagy flux, ammonium chloride (NH_4_Cl), an inhibitor of lysosome acidification, was used to inhibit lysosome degradation. PC12 cells were treated with or without NH_4_Cl (Greagent G17391B) 20 mM for 12 h during the reperfusion episode after IPC.

### siRNA treatment

Two small interfering RNA against GRP78 (siRNA1, sense: 5′-GAGGCGUAUUUGGGAAAGATT-3′, and antisense: 5′-UCUUUCCCAAAUACGCCUCTT-3′; siRNA2, sense: 5′-GCGUCGGUGUAUUCAAGAATT-3′, and antisense: 5′-UUCUUGAAUACACCGACGCTT-3′) were screened from six sequences designed and synthesized to target GRP78 by Genepharma Co. Ltd. (Shanghai, China). Transfection of GRP78 siRNA (40 nM) in PC12 cells was performed using lipofectamine 2000 (Invitrogen) according to the manufacturer’s instructions. The PC12 cells were also transfected with a control scrambled RNA targeting a sequence not sharing homology with the rat genome (negative control, NC). The suppression of GRP78 expression (after transfection for 36–48 h) was confirmed with Western blotting.

### Lentivirus-mediated GRP78 overexpression

LV5-Hspa5 rat-GFP-Puro (1 × 10^9^ TU/ml, Gene ID 25617, NM 013083) lentivirus was constructed by Genepharma Co.,Ltd. PC12 cells were infected with LV5-Hspa5 or LV-vector at multiplicities of infection (m.o.i.) of 10 for 48 h, and then 4 μg/ml puromycin (Sigma) was added to medium to kill the non-transfected cells. Twenty-four hours later, the cells were replaced with fresh medium containing 4 μg/ml puromycin. The GFP expression at 48 h was observed in >90% of the infected cells. Then the cells were selected with 4 μg/ml puromycin for 3 weeks. The stable GRP78 overexpressing PC12 cells were identified using GFP expression and Western blot analysis as the data showed.

### Statistical analysis

Significant differences between groups were determined with one-way ANOVA, and intergroup comparisons (post-hoc analysis) were carried out with the Newman-Keuls test.

## Additional files

Additional file 1: Figure S1. Immunofluorescent staining confirmed that LC3 and GRP78 were upregulated 12 h after IPC. The cells were stained with anti-GRP78 antibody (red), anti-LC3 (green) and DAPI (blue). Scale bar = 10 μm. Additional file 2: Figure S2. Suppression of GRP78 with two siRNA sequences inhibited autophagy activation. **(A)** GRP78 siRNAs deceased GRP78 expression in PC12 cells. Cells were transfected with GRP78 siRNA1 and siRNA2 (40 nM) or negative control sequence (NC) for forty-eight hours. **(B)-(C)** Cells were transfected with GRP78 siRNAs or NC twenty-four hours before the onset of IPC and then the cells were harvested 12 h after IPC. **(B)** GRP78 siRNAs reversed IPC-induced upregulation of GRP78. **(C)** GRP78 siRNAs reversed IPC-induced upregulation of LC3II/LC3I. Bar represents mean ± SD, n = 3. $ *P* < 0.05 compared with the NC group; ***P* < 0.01 compared with the control group; # *P* < 0.05, ## *P* < 0.01 compared with the NC + IPC group.

## Abbreviations

AMPK: AMP activated protein kinase
BAPTA: Dibromo-1,2-bis(aminophenoxy) ethane N,N,N9,N9- tetraacetic acid
CCK-8: Cell Counting Kit-8 assay kit
DAPI: 4,6-diamidino-2- phenylindole
DMEM: Dulbecco’s modified Eagle’s medium
ER: Endoplasmic reticulum
GRP78: Glucose regulated protein 78
Hsp70: Heat shock protein 70
IGFBP-3: Insulin-like growth factor binding protein-3
IPC: Ischemic preconditioning
mTOR: mammalian target of rapamycin
NBM: Neurobasal medium
NC: Negative control
LV: Lentiviral vector
OGD: Oxygen glucose deprivation
p70S6K1: p70 ribosomal protein S6 kinases 1
UPR: Unfolded protein response
