# Supplementary figures and images for: Endoplasmic reticulum chaperone GRP78 is involved in autophagy activation induced by ischemic preconditioning in neural cells
Source: Mol Brain. 2015 Mar 26;8:20. doi: 10.1186/s13041-015-0112-3 (PMC4381498; doi:10.1186/s13041-015-0112-3)

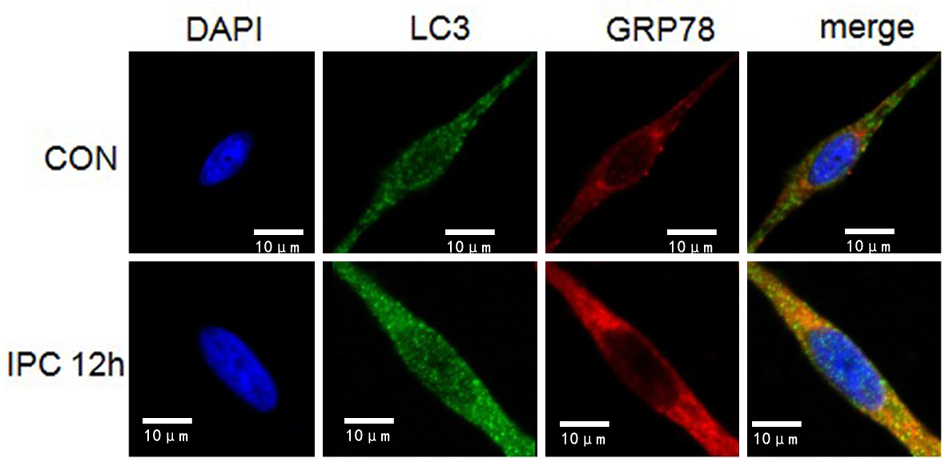

Supplement: Additional file 1: Figure S1. — Immunofluorescent staining confirmed that LC3 and GRP78 were upregulated 12 h after IPC. The cells were stained with anti-GRP78 antibody (red), anti-LC3 (green) and DAPI (blue). Scale bar = 10 μm. [file 13041_2015_112_MOESM1_ESM.tiff]

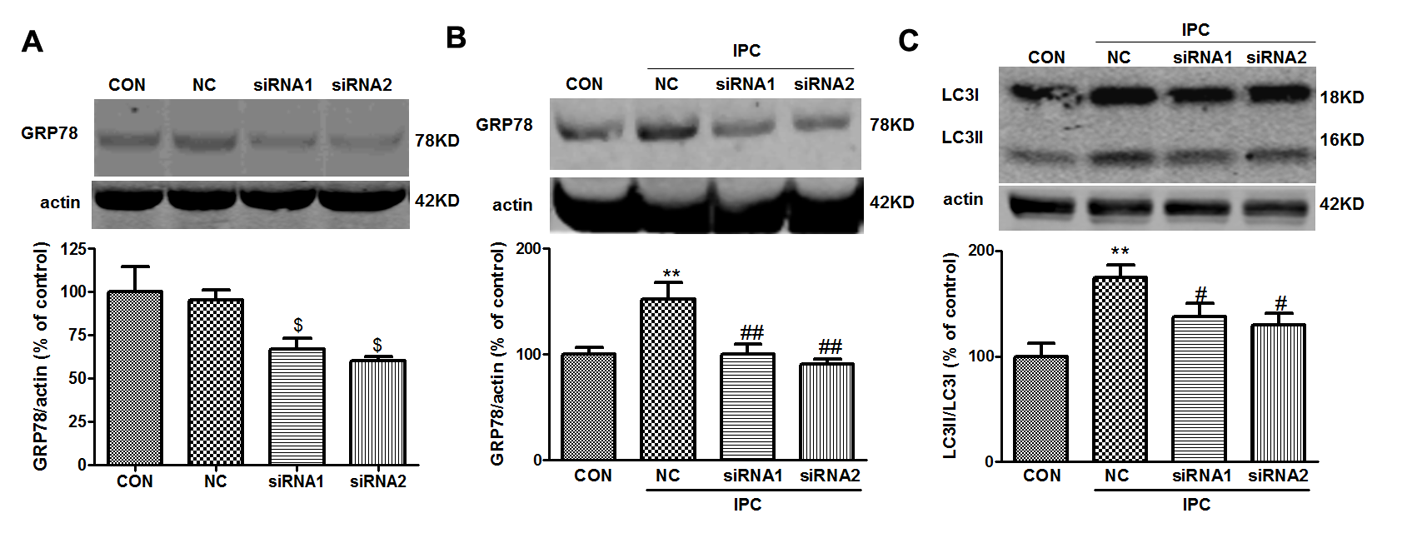

Supplement: Additional file 2: Figure S2. — Suppression of GRP78 with two siRNA sequences inhibited autophagy activation. (A) GRP78 siRNAs deceased GRP78 expression in PC12 cells. Cells were transfected with GRP78 siRNA1 and siRNA2 (40 nM) or negative control sequence (NC) for forty-eight hours. (B)-(C) Cells were transfected with GRP78 siRNAs or NC twenty-four hours before the onset of IPC and then the cells were harvested 12 h after IPC. (B) GRP78 siRNAs reversed IPC-induced upregulation of GRP78. (C) GRP78 siRNAs reversed IPC-induced upregulation of LC3II/LC3I. Bar represents mean ± SD, n = 3. $ P < 0.05 compared with the NC group; **P < 0.01 compared with the control group; # P < 0.05, ## P < 0.01 compared with the NC + IPC group. [file 13041_2015_112_MOESM2_ESM.tiff]
